# Coiled-Coil Structures Mediate the Intercellular Propagation of Huntingtin

**DOI:** 10.3390/ijms26178162

**Published:** 2025-08-22

**Authors:** Marco Bosica, Chiara Grasselli, Andrea Panfili, Franca Orsini, Luana Fioriti

**Affiliations:** 1 Istituto di Ricerche Farmacologiche Mario Negri IRCCS, 20156 Milano, Italy; marco.bosica@marionegri.it (M.B.); chiara.grasselli@marionegri.it (C.G.); andrea.panfili@marionegri.it (A.P.); franca.orsini@marionegri.it (F.O.); 2 Department of Cell Biology and Pathology, Columbia University, New York, NY 10032, USA

**Keywords:** huntingtin, prion-like propagation, aggregation

## Abstract

Huntington’s Disease (HD) originates from the expansion of a polyglutamine (PolyQ) tract in the huntingtin protein (Htt), which can assume a coiled-coil fold (Cc). We previously found that Cc structures mediate the aggregation and toxicity of polyQ Htt. Since polyQ Htt aggregates were previously found to be internalized by cells, here we hypothesize that Cc structures might be implicated in the intercellular propagation of Htt aggregates. To test this hypothesis, we performed experiments using human cell lines expressing Htt proteins with different probabilities to acquire a Cc fold. We found that Htt with reduced Cc structures were released significantly less compared to Htt with intact Cc structures. We also found that Cc structures mediate the internalization of Htt proteins in recipient cells. Together, these results underline the importance of the Cc structure in the process of intercellular propagation of Htt polyQ aggregates and suggest that interfering with Cc formation might be a therapeutic strategy for HD.

## 1. Introduction

Huntington’s disease (HD) is a debilitating neurodegenerative genetic disorder caused by the expansion of polyglutamine (polyQ) repeat in the huntingtin (Htt) gene [1,2,3]. In particular, this expansion occurs in the coding region of the first exon of the Htt gene (Htt exon 1). In physiological conditions, the polyQ region consists of a variable number of glutamines, from 9 to 34 residues, while under pathological conditions, the polyQ tract is longer than 35 residues of glutamine. The resultant pathological protein, mutant Htt (mHtt), undergoes misfolding and aggregation in neural cells, particularly affecting neurons in the striatum and cortex, leading to neuronal toxicity and cellular death [4].

Recent studies suggest that mHtt exhibits prion-like properties [5,6,7], capable of templating its misfolded state onto normal Htt and spreading between cells. In this respect, prion-like propagation has emerged as a unifying theory to explain the progressive and spatially distributed pathology observed in HD [6,8,9,10] and other neurodegenerative diseases. The first evidence of the prion-like propagation of mHtt originates from studies in vitro. For example, Ren et al. [7] showed that mHtt-expressing cells release aggregates that can be internalized by neighboring cells, inducing further aggregation of endogenous Htt. Seeding and templating activity in vitro studies with recombinant mHtt fibrils have shown that they can seed the aggregation of wild-type Htt. This templated misfolding is a hallmark of prion-like behavior and further supports the propagation hypothesis. Moreover, in vivo evidence was also obtained in animal models expressing mHtt in localized brain regions [6], which show the spreading of aggregates to distant areas over time, indicating a non-cell-autonomous mechanism of pathology. This supports the idea that mHtt can propagate through neural circuits [10,11].

Prion-like propagation of mHtt may explain the sequential and regional development of neuropathology in HD. It also raises the possibility that early focal aggregation events could trigger widespread neurodegeneration. Mechanistically, several pathways have been proposed to mediate the intercellular spread of mHtt: direct cell-to-cell contact and tunneling nanotubes [12,13,14], endocytosis of released aggregates, transport within exosomes [15], and other extracellular vesicles.

In previous work, we discovered that Htt exon 1 showed a propensity to form coiled-coil (Cc) structures [16]. A coiled-coil domain is a structural motif where 2–7 alpha-helices twist around each other [17,18]. This facilitates protein–protein interactions by promoting oligomerization and assembling protein monomers into complexes [19,20]. We found that Cc motifs promote oligomerization and self-association of mHtt, increase the likelihood of forming amyloid-like aggregates, and interact with other proteins through these domains, often sequestering essential cellular components [16]. Our initial findings were confirmed by other groups [21] and extended to other polyQ rich proteins [22,23]. Interestingly, Cc domains are present in a wide variety of proteins, and are often associated with membrane interactions [24,25]. Cc are also present in several proteins involved in the formation and release of exosome, such as Alix and Tsg101 [26]. Thus, we hypothesized that Htt Cc structures might promote the aberrant interaction of mHtt with these proteins and possibly contribute to the exosome-mediated intercellular propagation of mHtt. In the present study, we tested our hypothesis using human cell lines expressing Htt proteins with different probabilities to acquire Cc fold. We assessed their ability to interact with Alix, to be released from transfected cells, and to be internalized by recipient cells using a combination of techniques (Western blot, FACS, flow cytometry and confocal imaging). We found that Cc structures are critically involved in all these processes. Therefore, this study contributes meaningfully to the expanding literature on prion-like spread by identifying a structural determinant that regulates multiple steps in the life cycle of pathogenic mHtt.

## 2. Results

### 2.1. PolyQ Expanded Huntingtin Achieves the Aggregation Plateau at 48 H Following Transfection

**Figure 1:**
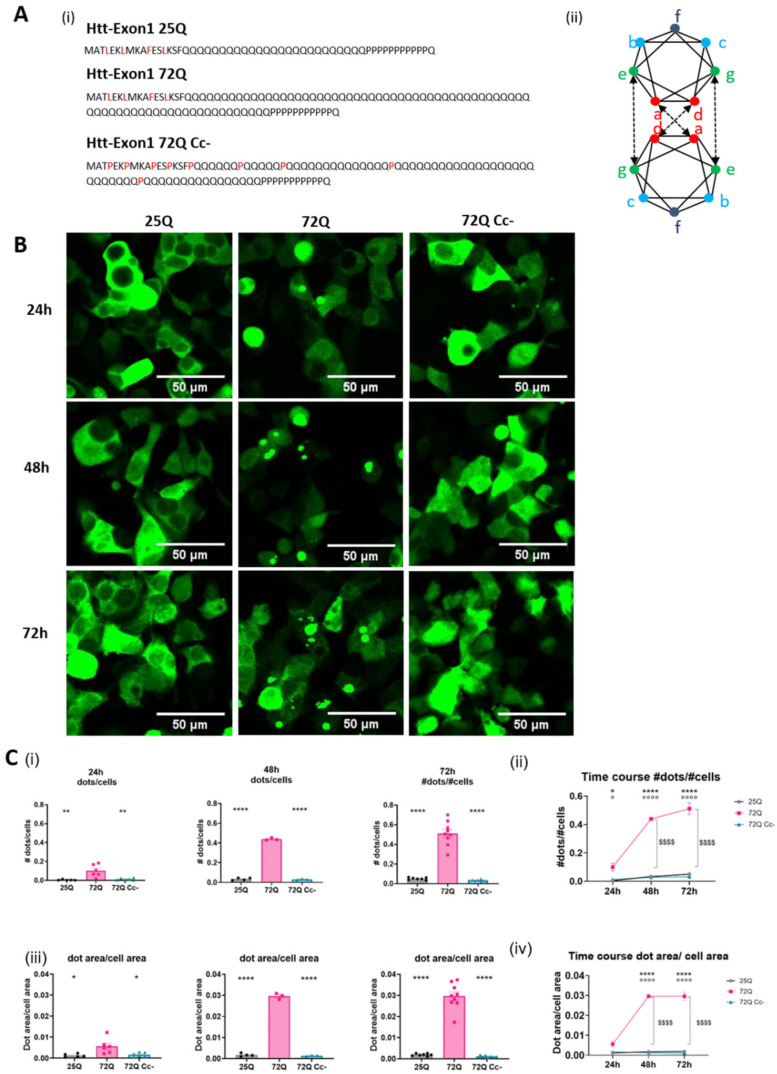
Time course of Htt aggregates formation. (**A**) (**i**) The aminoacidic sequences of the Htt constructs used: Htt-25Q, Htt-72Q, and Htt-72Q coiled-coil mutant. (**ii**) A schematic representation of zenithal view of two coiled helices. Red circles = heptad positions a/d, green circles = g/e, and cyan-to-blue circles = b, c and f. Aminoacids in position a,d are responsible for hydrophobic interactions between complementary coils (indicated with crossing arrows) while aminoacids in position e an g mediate electrostatic interactions between complementary coils (straight arrows). (**B**) Representative images of fluorescence micrographs of HEK293 cells overexpressing either Htt 25Q, 72Q, or its Cc defective mutants acquired with 20X magnification at three different time points (24 h, 48 h, and 72 h); scale bar: 50 μm. (**C**) Aggregation rate of the 3 Htt constructs. (**i**) Quantification of the number of aggregates per cell. The analyses were carried out at 24 h (n = 5–6), 48 h (n = 3–4), and 72 h (n = 8–9). One-way ANOVA followed by Tukey’s test; ** *p* < 0.001, **** *p* < 0.00001. (**ii**) Time course of the number of aggregates per cell. Two-way ANOVA followed by Tukey’s test; * *p* < 0.05, **** *p* < 0.00001 72Q vs. 25Q; ° *p* < 0.05, °°°° *p* < 0.00001 72Q vs. 72Q Cc-; $$$$ *p* < 0.00001 72Q 24 h vs. 48 h and 72 h. (**iii**) Quantification of the area occupied by aggregates on the area occupied by the transfected cells. The analyses were carried out at 24 h (n = 5–6), 48 h (n = 3–4), and 72 h (n = 8–9). One-way ANOVA followed by Tukey’s test; * *p*< 0.05, **** *p* < 0.00001. (**iv**) Time course of the area occupied by aggregates, normalized on the cell’s transfection area. The area of Htt 72Q dots increased at 48 and 72 h after transfection. Two-way ANOVA followed by Tukey’s test, **** *p* < 0.00001 72Q vs. 25Q; °°°° *p* < 0.00001 72Q vs. 72Q Cc; $$$$ *p* < 0.00001 72Q 24 h vs. 48 h and 72 h.

We previously reported that overexpression of mHtt fragments in HEK293 for 96 h induced the formation of large, toxic aggregates [16]. Here, we performed a time course analysis to better understand the kinetics of mHtt aggregation. We transfected HEK293 cells with constructs carrying Htt exon 1 fragments (Figure 1A(i)). We used Htt exon 1 with 25Q flagged with GFP (25Q), Htt exon 1 with 72Q flagged with GFP (72Q), or Htt exon 1 with 72Q coiled-coil–defective (Cc-) mutant, in which residues in a/d position (Figure 1A(ii)) have been substituted with prolines [27]. 72Q forms cytoplasmic aggregates which are visible starting at 24 h after transfection, while the 72Q Cc- has a diffused distribution, like the physiological construct 25Q (Figure 1B). We quantified (1) the number of dots (single aggregates) normalized on the number of cells (Figure 1C(i,ii)) and (2) the area occupied by dots normalized on the area of transfected cells (Figure 1C(iii,iv)).

We found that 24 h after transfection, the 72Q protein forms higher number and bigger dots per cell compared to 25Q (Figure 1C(i,iii)). In cells transfected with 72Q, the number and the size of the dots increase further at 48 and 72 h after transfection (Figure 1B,C). 25Q forms small aggregates with no increase in the number and in the dimension over time. 72Q Cc-, which has a reduced ability to form Cc structures, produces lower number and smaller aggregates compared to 72Q protein, like the 25Q protein did (Figure 1C). This confirms our previously published data that showed that the substitution of specific aminoacidic residues with prolines in 72Q (Figure 1A) can prevent aggregation of PolyQ-expanded huntingtin [16] and establish a kinetic profile of the aggregation of 72Q.

Different levels of aggregation prone proteins can potentially affect the aggregation process; therefore, to determine whether the different aggregation propensity of 72Q Cc- could originate from a reduced expression of the protein, we compared the levels of 25Q, 72Q, and 72Q Cc- by Western blotting. We found that 72Q Cc- is expressed at higher levels compared with 72Q (Supplemental Figure S1), thus confirming that the reduced aggregation is due to disruption of the Cc structure, and is not due to a decreased amount of protein available to form aggregates.

### 2.2. The Coiled-Coil Structure of Htt Exon 1 Mediates the Formation of Htt Insoluble Oligomers

**Figure 2:**
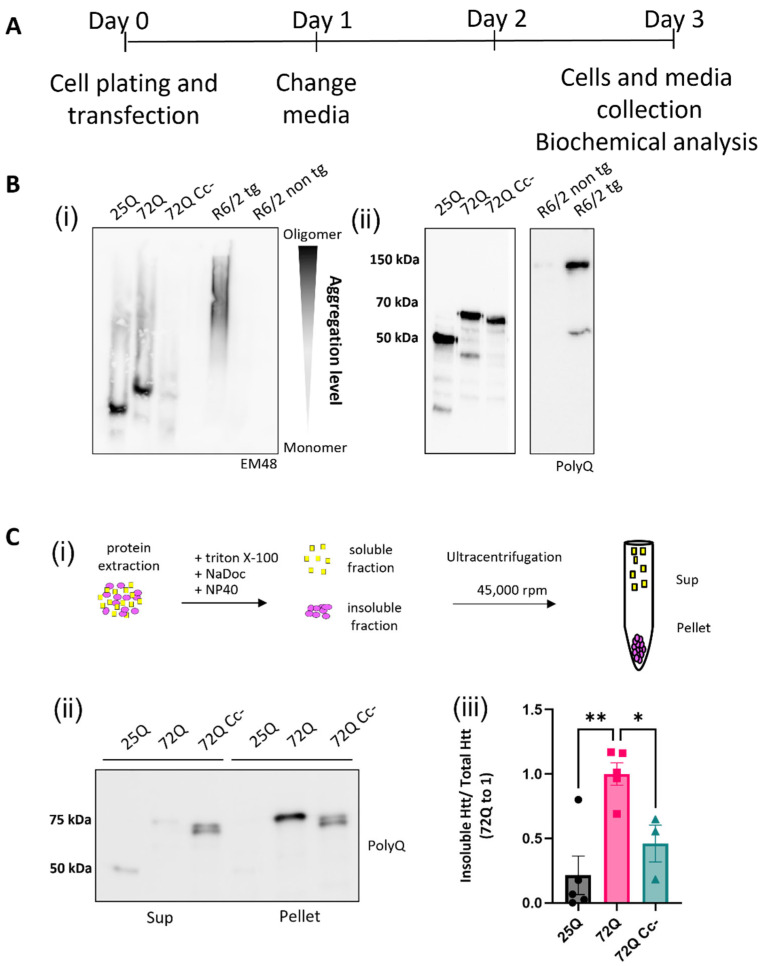
Coiled-coil abolition reduces aggregation and insolubility of Htt exon 1 72Q. (**A**) Experimental design. (**B**) (**i**) Representative image of Htt constructs aggregation via Semi-Denaturing Detergent Agarose Gel Electrophoresis (SDD-AGE). As positive control, we used R6/2 transgenic mice brain samples, overexpressing the first exon of the human mutant huntingtin gene with approximately 160 CAG repeats, and their non-transgenic littermate as negative control. (**ii**) Representative image of WB analysis to demonstrate equal expression. (**C**) (**i**) Schematic representation of detergent insolubility assay obtained by ultracentrifugation. (**ii**) Western blots of ultracentrifugation assays on lysates of HEK293 cells overexpressing 25Q, 72Q, or 72Q Cc defective mutant. Soluble (S) and insoluble pellet (P) fractions were incubated with primary antibody selective for Poly-Q sequence. (**iii**) Quantification of the pellet insoluble fraction over the total (S + P) Htt protein in ultracentrifugation assay. 72Q was enriched in the insoluble fraction compared to the soluble one; 25Q and 72Q Cc- mutants displayed opposite pattern with a remarkable reduction in the insoluble fraction. One-way ANOVA, followed by Tukey’s test * *p* < 0.05, ** *p* < 0.01. Bars represent mean ± SEM (n = 3–5 from three independent experiments).

To biochemically characterize the aggregation of Htt exon 1 fragments, we performed Semi-Denaturing Detergent Agarose gel electrophoresis (SDD-AGE) on HEK293 lysates 72 h after transfection (Figure 2A). With this technique, polymeric and insoluble proteins can be visualized as high molecular weight species compared to the monomeric protein [28,29,30]. Brain homogenates from 14-week-old B6CBA-R6/2 transgenic mice, carrying 160 CAG repetitions [31], and their non-transgenic littermates were also analyzed as controls. We found that 72Q protein forms large aggregates, which appear as a smear in the gel, above the expected size of the monomeric protein. This aggregation profile was similar to 160Q Htt expressed by R6/2 transgenic mice (Figure 2B(i)). 25Q and 72Q Cc- proteins, on the other hand, did not form large aggregates. The difference cannot be explained by a different amount of protein, as equal loading was confirmed in regular SDS-PAGE blots (Figure 2B(ii)). To exclude the possibility that the absence of signal in the 72Q Cc- samples is due to masking of the epitope in the 72Q Cc- protein, we probed the samples with additional antibodies, directed against different portions of the protein, and obtained the same results (Supplemental Figure S2), thus confirming that 72Q Cc- does not form large aggregates. Aggregated proteins can be insoluble to certain detergents [16,28,30,32,33]. This biochemical property can be studied in cell lysates using ultracentrifugation to separate detergent-insoluble aggregates from the soluble species. We collected lysates of HEK293 cells overexpressing Htt variants for 72 h treated with nonionic detergents and performed ultracentrifugation experiments (Figure 2C(i)). We found that 72Q was enriched in the insoluble fraction compared to the soluble one (Figure 2C(ii)). On the contrary, 25Q and 72Q Cc- mutants displayed opposite pattern with a remarkable reduction in the insoluble fraction. The insolubility was quantified in three independent experiments and like 25Q, the 72Q Cc- showed a significant reduction in protein insolubility compared to 72Q (Figure 2C(iii)). Thus, detergent insolubility correlated with Cc propensity, closely paralleling the aggregation phenotypes.

### 2.3. Htt Exon 1 Colocalizes with Alix

**Figure 3:**
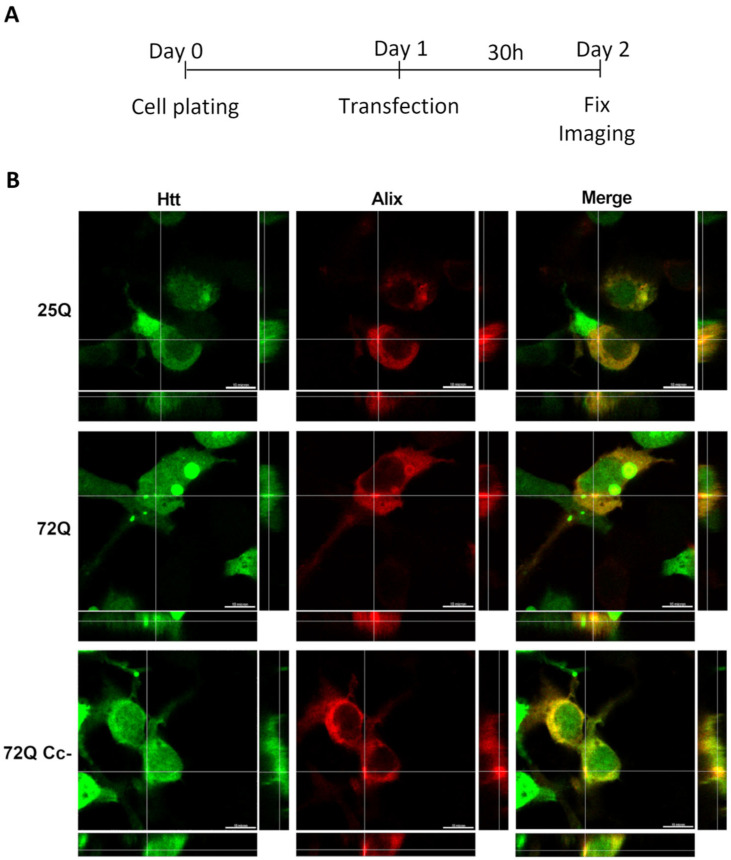
Htt 72Q colocalizes with exosome marker Alix. (**A**) Experimental design. (**B**) Representative images of Htt exon 1 constructs (green) co-transfected with exosomal marker Alix (red), acquired 30 h after transfection using 100× objective. Alix signal was diffused throughout the cytosol but it also colocalized with 72Q aggregates. In cells transfected with 25Q and 72Q Cc-, the Alix signal was more diffused. Scale bar: 10 μm.

Next, we performed co-transfection experiments to evaluate the co-presence of Htt exon 1 proteins with an exosomal marker. To do this, we transfected HEK293 cells with plasmids encoding the 25Q, 72Q, and 72Q Cc- Htt exon 1 fragments together with a plasmid encoding Alix, ALG-2 interacting protein X, a cytoplasmic protein present on exosomal membranes [34,35,36] (Figure 3A). We used a plasmid expressing Alix fused with red fluorescent protein (RFP) in the carboxy-terminal portion to visualize it. We confirmed that the transfected Alix-RFP had a distribution similar to the endogenous protein (Supplemental Figure S3). After 30 h of transfection, we observed that Alix generally had a diffused distribution, similar to the endogenous protein (Supplemental Figure S3), but with areas where the signal was more concentrated. In cells transfected with 72Q, we observed a colocalization between Alix and 72Q aggregates as well as in their close proximity, as shown in Figure 3B. However, when Cc structures are impaired, this phenomenon was reduced. These results suggest that Cc structures may be implicated in the release of Htt, by promoting the interaction with exosome-associated proteins.

### 2.4. Htt 72Q Secretion Is Reduced When the Coiled-Coil Structure Is Hampered

**Figure 4:**
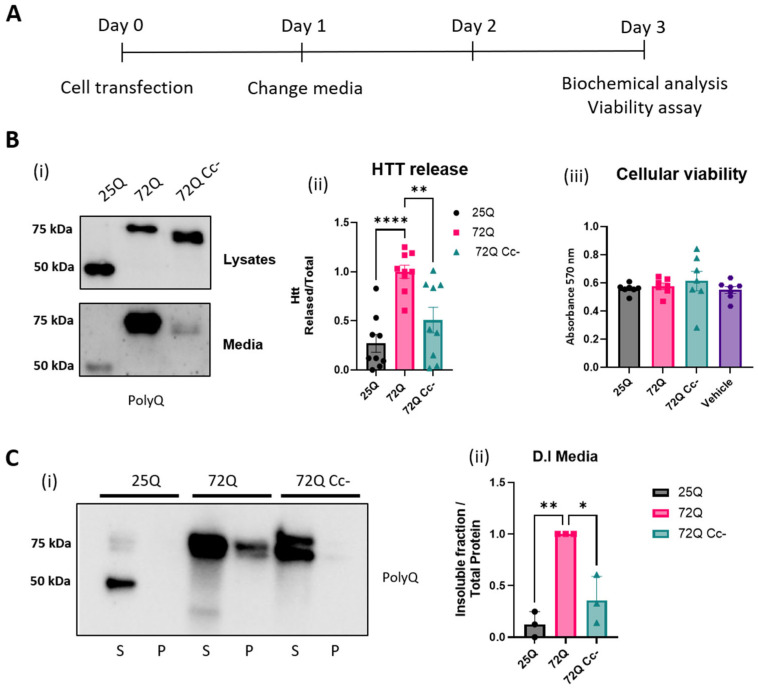
Htt 72Q is released in insoluble form in culture media, and its secretion is reduced if the coiled-coil structure is hampered. (**A**) Experimental design. (**B**) (**i**) Representative WB images of lysates and cell media incubated with primary antibody selective for the sequence Poly-Q. (**ii**) Quantification of the proteins released in the media, in relation to the total Htt expression (lysate + media). All Htt proteins were released in the medium. 72Q protein is released significantly more compared to 25Q and 72Q Cc-. One-way ANOVA followed by Tukey’s test, ** *p* < 0.01 **** *p* < 0.0001 (n = 7–9 replicates from 3 independent experiments). Bars represent mean ± SEM. (**iii**) Alamar Blue viability test carried out at 72 h post transfection showed that expression of the constructs for up to 72 h did not induce cellular toxicity. One-way ANOVA followed by Tukey’s test. No significant difference between the groups was found. Bars represent mean ± SEM. (**C**) Quantification of Htt insoluble fraction released in the media. (**i**) Western blot of DI assays on the media of HEK293 cells overexpressing 25Q, 72Q, or 72Q Cc defective mutant. Soluble (S) and insoluble pellet (P) fractions were incubated with anti Poly-Q antibody. (**ii**) Quantification of DI assays. 72Q released in the medium was significantly more insoluble compared to both 25Q and 72Q Cc- (n = 3–5 from three independent experiments). One-way ANOVA followed by Tukey’s test, * *p* < 0.05 ** *p* < 0.01. Bars represent mean ± SEM (n = 3 from three independent experiments).

Next, we conducted biochemical analyses to determine more directly a possible involvement of Cc structures in the release of Htt proteins in the media of HEK293 transfected cells. As shown in the experimental design (Figure 4A), 24h after transfection, the media was replaced to remove plasmid DNA. Forty-eight hours later, the media was collected, and the cells were harvested and lysed. Proteins in the media were concentrated and loaded for SDS-PAGE Western blot (Figure 4A) along with protein cellular extracts. We found that all Htt proteins were released in the medium, as shown in Figure 4B(i). The ratio of released protein to intracellular protein was calculated (Figure 4B(ii)) and we found that 72Q protein was released significantly more into the media compared to 25Q. We also found that the release of the 72Q Cc- was significantly lower than that of 72Q. Our data, therefore, suggest that the presence of the extended polyQ sequence in mHtt leads to an increase in its release, and that this phenomenon is, at least in part, dependent on the presence of an intact coiled-coil structure in 72Q.

To further establish a link between the Cc structure, aggregation, and release of Htt exon 1 fragments, we transfected HEK293 cells with a construct expressing 25Q with an enhanced propensity to form coiled-coil structures, named 25Q+ (Supplemental Figure S4A). We found that 25Q+ forms aggregate in transfected HEK293 cells, and it is released five times more compared with 25Q. Moreover, to confirm that this phenomenon is not unique to HEK293 cells, but can be extended to neuronal cells, we expressed the same construct in SH-SY5Y cells and confirmed that 25Q+ forms aggregates (Supplemental Figure S4B) and is released more than 25Q Htt (Supplemental Figure S4C).

Since mutant forms of Htt can be toxic to cells, and dying cells can release their cytosolic content into the surrounding extracellular space, to exclude that the presence of mHtt in the media was due to release from dying cells, we performed an Alamar blue viability assay. We found that expression of the constructs for up to 72 h did not induce any evidence of cellular toxicity (Figure 4B(iii)), confirming that the presence of Htt fragments in the culture media was likely due to an active process of protein release from living cells. Next, we evaluated the solubility of the released proteins by D.I. analysis. Given the difference in the amount of protein released, we balanced the volume of each sample in order to have the same amount of protein for each condition and separated the soluble protein fraction from the insoluble fraction. The soluble and insoluble fractions were analyzed by Western blot (Figure 4C(i)). The quantification of the insoluble Htt showed that 72Q had significantly higher values compared to both 25Q and 72Q Cc- (Figure 4C(ii)), similarly to what we found in cellular extracts (Figure 2C(iii)). These results highlight the relevance of Cc structure in determining the biophysical properties of Htt.

### 2.5. Htt 72Q Uptake Is Reduced When the Coiled-Coil Structure Is Hampered in Cell-to-Cell Contact Conditions

**Figure 5:**
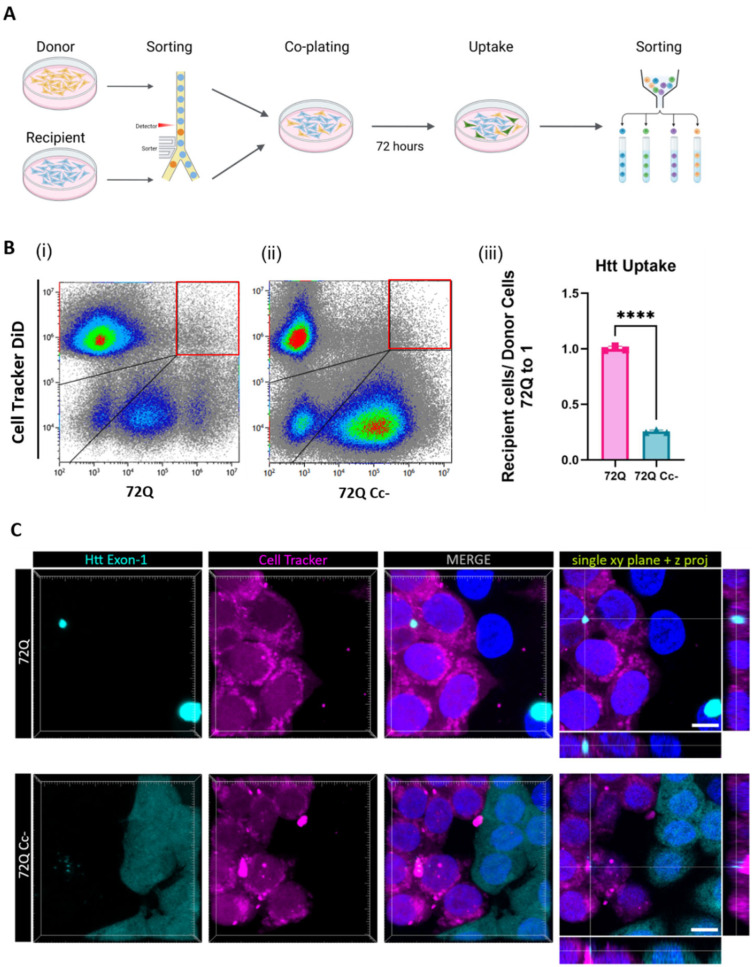
Uptake of Htt in co-culture experiments. (**A**) Experimental design. Hek293 cells expressing 72Q or 72Q Cc- and HEK293 cells labeled with DiD tracker were sorted and co-plated 1:1. (**B**) Detection of Htt exon 1 fragments in DiD Cell Tracker labeled recipient cells by flow cytometry. (**i**) Positive counts 72Q RFP and Cell Tracker DiD far red signal, (**ii**) positive counts 72Q Cc-GFP and Cell Tracker DiD far red signal. Both 72Q and 72Q Cc- proteins were internalized by recipient cells. (**iii**) Statistical analysis of positive recipient cells on donor cells obtained from 3 independent experiments (n = 3 per condition) showing that the internalization of the 72Q Cc- protein in recipient cells was significantly reduced compared to 72Q in cell-to-cell contact conditions. One-way ANOVA followed by Tukey’s test, **** *p* < 0.0001. Bars represent mean ± SEM. (**C**) Hek293 cells expressing Htt exon 1 72Q or 72Q Cc- and Cell Tracker labeled cells were sorted and co-plated in the ratio 1:1 after 72 h in co-plating cells were fixed. Images are acquired with 60× objective. Both 72Q and 72Q Cc- proteins can be internalized by recipient cells, as shown by x, y single-plane image with z-projections. Scale bar: 10 µm.

After we established the role of the Cc structure in the release of the Htt exon 1 fragment, we investigated the role of Cc in the process of uptake from recipient cells. In the following experiments, we focused our attention on the 72Q and 72Q Cc- defective mutant. We transfected HEK293 cells with Htt constructs (donor cells), and 24 h later we co-plated them with cells labeled with cell Tracker DiD (recipient cells) (Figure 5A). We chose this dye because once incorporated into cells, it is retained at least for 72 h (Supplementary Figure S5). Donor and recipient cells were sorted by Fluorescence-activated cell sorting (FACS) in order to co-plate the same amount of donor and recipient cells in each condition. After 72 h in co-culture, cells were harvested and fluorescence was assessed by flow cytometry (Figure 5B(i,ii) and Supplementary Figure S6). Our analysis allowed us to identify four populations: Htt exon 1 positive (lower-right box in figure), DiD positive (upper-left box in figure), Htt exon 1/DiD double positive (upper-right box in figure), and double negative (lower-left box in figure). The double positive cells represent recipient cells, initially labeled only with DiD, that internalized Htt exon 1 during the 72 h co-plating time. We found that both 72Q and 72Q Cc- proteins can be internalized by recipient cells. However, the internalization of the 72Q Cc- protein in recipient cells was significantly reduced compared to 72Q under these conditions (Figure 5B(iii)). The results were also confirmed by confocal imaging analyses (Figure 5C).

### 2.6. Coiled-Coil Structure Promotes the Uptake of 72Q Htt Present in the Medium

**Figure 6:**
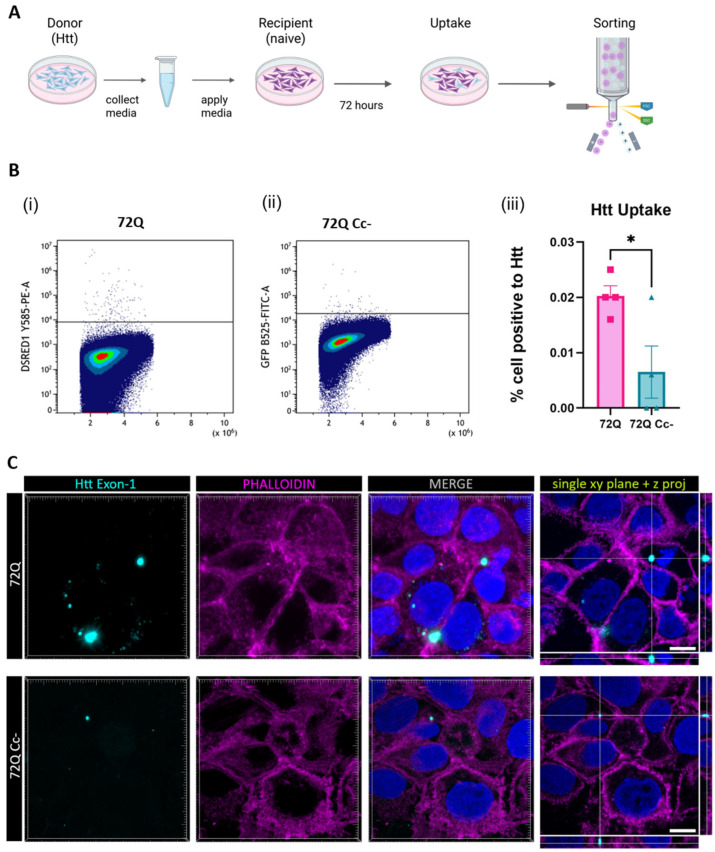
Uptake of Htt in cells exposed to media containing Htt. (**A**) Experimental design. 72 h after transfection with 72Q or 72Q Cc-, culture media was collected and the amount of Htt was quantified by Western blot analysis. Hek293 naïve cells were exposed for 96 h to conditioned media containing the same amount of 72Q or 72Q Cc-. (**B**) Detection of Htt in recipient cells by flow cytometry analysis. (**i**) Cells positive for 72Q, (**ii**) cells positive for 72Q Cc-. (**iii**) Statistical analysis of positive recipient cells obtained from 4 independent experiments (n = 4 per condition). One-way ANOVA followed by Tukey’s test, * *p* < 0.05. Bars represent mean ± SEM. The internalization of the 72Q Cc- protein in recipient cells was significantly reduced compared to 72Q after exposure to conditioned media. (**C**) Confocal images of cells exposed to media collected from 72Q or 72Q Cc- transfected cells. Cells were fixed after 96 h of incubation with the media and stained with Phalloidin dye. Images were acquired with 100× objective. Both 72Q and 72Q Cc- proteins can be internalized by recipient cells as shown by x, y single-plane image with z-projections, however 72Q was internalized more than 72Q Cc-. Scale bar: 10 µm.

The greater ability of 72Q to be internalized by recipient cells might be partially due to the fact that it is released in greater amount (Figure 2) compared to 72Q Cc- defective mutant. To exclude this possible confounding effect, we performed another set of experiments in which recipient cells were exposed to media containing the same amount of 72Q or 72Q Cc (Figure 6A).

To achieve this, first cells were transfected with 72Q or 72Q Cc. Twenty-four hours after transfection, the media was replaced and cells washed, to remove any possible trace of plasmid DNA. A total of 72 h after media replacement, conditioned media were collected, and the amount of Htt fragments were quantified by Western blot. This allowed us to estimate the volume of conditioned media derived from cells transfected with 72Q or 72Q Cc to be used from the different samples before applying them to naive recipient cells. A total of 96 h after exposure to the media, cells were harvested and fluorescence was assessed by flow cytometry analysis. We found that cells internalized 72Q significantly more than 72 Cc- (Figure 6B). Confocal analysis (Figure 6C) further demonstrated the internalization of Htt proteins contained in the media by naïve cells.

These results, together with previous results in cell-to-cell contact conditions, suggest that the coiled-coil structure directly influences both the release of the Htt fragments and the uptake process by recipient cells.

## 3. Discussion

This study explores the role of polyglutamine (PolyQ) expansion and coiled-coil (Cc) structural elements in the aggregation, secretion, and intercellular transfer of mutant huntingtin (mHtt) exon 1 fragments, using HEK293 cells as a model system. The investigation builds on our prior findings that the overexpression of mHtt fragments results in large, toxic aggregates forming over time [16]. In this work, we focused on understanding the kinetics of mHtt aggregation and the molecular determinants driving its spread between cells.

The concept that mutant huntingtin (mHtt) might spread from cell to cell in a prion-like fashion has gained substantial traction over the past decade [5,8,15], drawing parallels to what has been observed in diseases like Alzheimer’s, Parkinson’s, and ALS. Prion-like propagation describes the phenomenon by which misfolded proteins can act as seeds, converting normally folded counterparts into pathological conformers, thereby enabling the spread of pathology across brain regions [13,37,38].

Early foundational work by Ren et al. (2009) [7] and Pecho-Vrieseling et al. (2014) [10] provided the first experimental evidence that mHtt can be transmitted between neurons and from neurons to glia, with consequent toxicity. These studies laid the groundwork for the idea that mHtt aggregates can move beyond their cell of origin, contributing to Huntington’s disease’s progressive, region-specific neurodegeneration [5,39,40].

Subsequent research, such as Pearce et al. (2015) [41] and Babcock & Gan (2021) [6], explored potential mechanisms for this intercellular transfer [42]. They proposed pathways involving tunneling nanotubes, synaptic transmission, and, more recently, extracellular vesicle (EV) such as exosomes. Studies by Jeon et al. (2016) [15] and Trajkovic et al. (2017) [43] demonstrated that mHtt can be incorporated into exosomes and released into the extracellular space, where it may be taken up by neighboring cells, suggesting a role for the endosomal–lysosomal system in disease spread. Our data on the colocalization between mHtt and Alix, a marker of exosomes, further suggest that mHtt might be released by exosome by interacting with critical components of the exosome machinery.

Our study builds on this body of work by identifying a key structural motif—the coiled-coil (Cc) domain—as a critical mediator of this prion-like behavior [44]. While many prior studies focused on polyQ length as the primary determinant of aggregation and toxicity, our work adds a new layer by showing that the Cc structure actively facilitates aggregation, secretion, and uptake of mHtt. This structural insight refines our understanding of what makes particular Htt species more prone to propagation. Similar results were obtained with other prion-like Cc containing proteins [23].

Importantly, this study uses a Cc-deficient mutant of Htt-72Q to show that disruption of this domain impairs nearly every step of the prion-like process: aggregation, association with exosomal machinery (via ALIX), secretion into the medium, and uptake by neighboring cells. We further demonstrate that increasing Cc propensity within the non-expanded 25Q Htt (25Q+) induces aggregation and release from non-neuronal, as well as neuronal cell lines. The use of both cell-contact and conditioned media experiments confirms that the Cc motif enhances intracellular aggregation and functions as a facilitator for efficient intercellular trafficking.

This ties in with growing interest in the biophysical properties of aggregating proteins, such as their ability to form specific interaction domains [20,21,22,45], undergo phase separation [46,47], or hijack vesicular transport systems. For instance, similar motifs have been implicated in tau and α-synuclein propagation. Thus, this study positions mHtt—specifically its Cc structure—within the broader framework of molecular features driving pathogenic spread.

Recent evidence indicates that low-complexity regions (LCRs) in prion-like domains often overlap with coiled-coil sequences, a feature likely critical for their function [23,44]. In humans, prion-like proteins are important regulators of transcription and are linked to diseases ranging from neurodegeneration to cancer. Although few functional prions have been well characterized [28,29,30] compared to pathological amyloid proteins, common traits are emerging. These proteins typically separate their functional globular domains from prionic regions, with prionic traits concentrated in LCRs that tend to form coiled coils and mediate protein–protein interactions, either directly or upon binding [48]. Many prion-like proteins also bind DNA or RNA through their globular domains, contributing to liquid–liquid phase separation and the formation of membraneless compartments. While toxicity is often attributed to the intrinsic harmfulness of aggregates, recent findings suggest an alternative view: cytotoxicity may stem from mislocalization, loss of normal interactions, or aberrant signaling caused by abnormal clustering. Ccs play a key role in these processes [17], as pathogenic mutations frequently alter their helical propensity.

Despite significant progress, major questions remain. It is unclear how prion-like proteins regulate transcription under specific conditions, what triggers their amyloid conversion in vivo, and how conformational changes in prion-like domains drive amyloid formation. A model in which Cc-mediated transitions enable functional-to-pathogenic conversion provides a plausible explanation. Demonstrating such transitions at the molecular level could reveal new mechanisms of transcriptional regulation and clarify why these proteins are consistently implicated in human disease.

In this context, our research opens up the possibility that disrupting the Cc structure might mitigate the cell-to-cell transmission of mHtt [49]. While earlier strategies focused on silencing or degrading the mutant transcript, this work suggests that blocking intercellular propagation could be an effective adjunct or alternative approach.

## 4. Materials and Methods

### 4.1. HEK 293 and SH-SY5Y Cell Culture

Cells were cultured in Dulbecco modified medium (DMEM) with 10% fetal bovine serum (FBS) and 100 U/mL penicillin–streptomycin and incubated at 37 °C with 5% CO_2_.

### 4.2. PC12 Cell Culture

PC12-Htt74Q cells (refs) were cultured in Dulbecco modified medium (DMEM) with heat inactivated horse serum (10%) tetracycline-free FBS (5%), penicillin–streptomycin (100 U/mL), Hygromycin (75 µg/mL) and geneticin (100 µg/mL) and incubated at 37 °C with 10% CO_2_. Htt expression was induced replacing the entire media with fresh media containing Doxycycline (1 µg/mL). A total of 48 h after Doxycycline induction, media culture was discarded and cells were harvested in ice-cold PBS 1X

### 4.3. Plasmids and Transient Transfection

The Htt plasmid used in this work were previously described [16]. pDEST-EGFP-Htt25Q-Exon1 encodes the physiological human Htt exon 1, which contains 25 Glutamines (25Q), pDEST-EGFP-Htt25Q-Exon1 with increased propensity to form Cc (25Q+); pDEST-EGFP-Htt72Q-Exon1 and pDEST-RFP-Htt72Q-Exon1 were used to express a pathological form of human Htt that contains an expansion of the PolyQ stretch to 72 Glutamines (72Q). pDEST-EGFP-Htt72Q-Exon1-Cc- was used to express the Htt pathological sequence (Htt72Q-Exon1) (Figure 1A(i)) with reduced Cc propensity (72Q Cc) [16] (Figure 1A(ii)). ALG-2 interacting protein X (Alix) plasmid was also used in co-transfection experiments (Addgene #21504).

Cells were transfected using Lipofectamine™ 3000 (#L3000-015, Invitrogen, Waltham, MA, USA) diluted in serum-free media. The following quantities of total DNA were used: 250 ng/well for the µ-Slide 8 Well imaging chamber; 500 ng/well for the 24-well culture plate; 1 µg/well for the 12-well culture plate; and 1.5 µg/well for the 6-well culture plate.

### 4.4. Viability Assays

The cell’s viability was detected using Alamar Blue (DAL1025, Invitrogen, Waltham, MA, USA) 72 h after transfection. The fluorescence intensity was quantified with the spectrophotometer TECAN plate reader M200, using λ excitation of 560 nm and λ emission of 590 nm.

### 4.5. Biochemical Assays

#### 4.5.1. Cell Lysis and Protein Extraction

Cells were lysed in extraction buffer (50 mM Tris-HCl [pH 7.5], 50 mM KCl, and 10 mM MgCl_2_) supplemented with complete protease and phosphatase inhibitor cocktails (Roche Applied Science, Basel, Switzerlan). Cellular debris were removed by centrifugation at 7000× *g* rpm for 10 min. The proteins contained in the media were precipitated with four volumes of methanol overnight at −20 °C and separated by centrifugation at 13,000 rpm for 30′ at 4 °C.

#### 4.5.2. Detergent Insolubility (D.I)

D.I was carried out adding 20 μL of D.I buffer 2X (1% Triton, 1% NP 40, 1% Sodium deoxycholate (SOD), 20 mM Tris-HCl- pH 7.5 In PBS 1X) to 20 μL of cell extracts and incubated for 30′ at 4 °C in continuous rotation. After rotation, samples were centrifuged at 45′000 rpm for 45′ at 4 °C. The pellets (P) were collected and the proteins of the supernatants (S) were precipitated with four volumes of methanol overnight at −20 °C and separated by centrifugation at 13,000 rpm for 30′ at 4 °C. Both S and P fractions were resuspended in Laemmli Sample Buffer (LMSB)-2X with 100 mM Dithiothreitol (DTT), boiled for 10′ at 95 °C at 300 rpm, and loaded in a 10% acrylamide gel.

D.I was also carried out on Htt proteins released in the media. Collected Media was incubated with 10X D.I buffer (5% Triton, 5% NP 40, 5% Sodium deoxycholate (SOD), 100 mM Tris-HCl- pH 7.5 In PBS 1X) at 4 °C in continuous rotation for 30 min and then centrifuged at 45′000 rpm for 45′ at 4 °C. The pellets (P) were collected and the proteins of the supernatants (S) were precipitated with four volumes of methanol overnight at −20 °C and separated by centrifugation at 13,000 rpm for 30′ at 4 °C. At the end of the procedures total protein homogenates and D.I. samples were suspended LMSB-2X with 100 mM Dithiothreitol (DTT), boiled for 10′ at 95 °C at 300 rpm. Protein samples were then loaded in a 10% acrylamide using Laemmli running buffer (192 mmol/L Tris-base, 78 mmol/L Tris-base, 0.1% SDS) and transferred on PVDF membranes using a semidry Trans-Blot^®^ Turbo™ Transfer System (Bio-Rad, Hercules, CA, USA) with the mixed molecular weight program for mini membranes (1.3 A, 25 V, 7 min)

#### 4.5.3. Semi-Denaturing Detergent Agarose Gel Electrophoresis (SDD-AGE)

Protein samples from in vitro experiments were obtained as described above and mixed with agarose loading buffer 2x (150 mM Tris-HCl pH 6.8, Sodium dodecyl sulfate (SDS) 2% Glycerol 33%, Bromophenol Blue 0.025%). Protein extracts from cortical tissues of R6/2 transgenic and non-transgenic littermates were obtained by homogenization in 10 volumes (*w*/*v*) of tris-saline (100 mmol/L Tris, pH 7.4, 150 mmol/L NaCl) supplemented with complete protease and phosphatase inhibitor cocktails (Roche Applied Science). Samples were further sonicated by 10 ultrasound pulses with a Branson sonifier. The homogenates were then mixed in agarose loading buffer 2x, and samples are loaded in 1.5% agarose gel containing 375 mmol/L Tris–HCl, pH 8.8, and SDS 0.1%. Samples were run in SDD-Age running buffer (20 mM Tris-base, 200 mM Glycine and 0.1% SDS).

After the electrophoretic run, proteins were transferred on PVDF membranes using wet tank transfer system at 100 V for 90′ with a Biorad 200 power supply (transfer buffer: 20 mM Tris-base, 200 mM Glycine 0.1% SDS, 15% methanol). All the membranes were blocked in 3% BSA for 1 h at RT and then probed with primary antibodies (see Table 1) overnight at 4 °C, followed by HRP secondary antibodies (see Table 2). Western blot images were acquired with a ChemiDoc MP Imaging System (Bio-Rad, USA) and quantified using the Image Lab software 6.0.

### 4.6. Huntingtin Uptake in Co-Plating Conditions

Donor cells were transfected with Htt exon 1 constructs and cultured for 24 h. Recipient cells were labeled with Cell Tracker deep red (C34565, Invitrogen, Waltham, MA, USA). Hek 293 Cells (10^6^ cells/mL) were incubated for 1 h with the dye using the final concentration of 25 μM in serum-free DMEM. The tubes were inverted in the middle of the protocol to guarantee proper mixing during the incubation. After incubation, cells were centrifuged 5′ at 1000 rpm and the supernatant was replaced with DMEM Phenol-Red Free (31053028, Thermo Fisher Waltham, MA, USA). After staining, both donor and recipient cells were sorted, and the positive cells were plated at a ratio of 1:1 and cultured for additional 72 h, after which cells were harvested in D-PBS and analyzed through flow cytometer.

### 4.7. Huntingtin Uptake Through Conditioned Media

Hek 293 transfected cells with 72Q and 72Q Cc- were cultured for 72 h, after which media was collected for biochemical assays to quantify the amount of Htt released in the media. Next, equal amounts of each protein were applied to naive Hek 293 cells for 96 h.

### 4.8. Cell Sorting

FACS was carried out on a MoFlo Astrios cell sorter (Beckman Coulter, Miami, FL, USA), using an average sorting rate of 2500–3000 events per second at a sorting pressure of 25 psi with a 100 μm nozzle was maintained. Cells were identified by size and granularity using FSC-A versus SSC-A. Single cells were identified and gated using FSC-A versus FSC-H. The cells with the highest fluorescence intensity of GFP (72Q Cc), RFP (72Q), or far red (Cell Tracker labeled cells) were isolated. Post-sort analysis was performed using Kaluza 1.2 software (Beckman Coulter, Miami, FL, USA).

### 4.9. Flow Cytometry Analysis

Cells were analyzed with Cytoflex LX flow cytometer equipped with CytExpert Acquisition 2.3 software (Beckman Coulter, Miami, FL, USA) based on fluorescence intensity. The cells with the highest fluorescence intensity of GFP (72Q Cc), RFP (72Q), and far red (Cell Tracker labeled cells) were assessed. The acquisition process was stopped when all events were collected in the population gate. Offline analysis was performed using Kaluza 1.2 software (Beckman Coulter, Miami, FL, USA). A conventional gating strategy was used to remove aggregates, and the percentages of GFP and/or RFP positive cells were quantified.

### 4.10. Immunofluorescence Assays

Media was removed, cells were washed with room temperature PBS, and cells were fixed in 4% Paraformaldehyde and 4% Sucrose in PBS for 10′.

After 3 washes with PBS, cells were permeabilized 5 min with PBS 0.5% Triton and blocked 1 h with PBS 5% NGS. Cells were incubated with Phalloidin Alexa fluor 647 conjugated (1:20 dilution in PBS) for 20 min. After 3 washes with PBS, cells were incubated with DAPI (1 μg/mL) for 10′ and left in PBS until confocal microscope acquisition.

### 4.11. Confocal Microscope Acquisition

Images were acquired using a confocal A1 system (Nikon, Tokyo, Japan) equipped with a confocal scan unit with 405 nm, 488 nm, 561 nm, and 640 nm laser lines with a scanning sequential mode to avoid bleed-through effects. For Htt granules quantification images were acquired using a 20× objective. For Htt internalization higher-magnification images were acquired using a 60× or 100× objectives over a 9- or 4.5-μm z axis with a pixel size of 0.21 μm or 0.12-μm and processed by using Imaris software 7.4.2 (Bitplane). Three-dimensional acquisitions were displayed as volumes and as x, y single-plane image with z-projections.

### 4.12. Huntingtin Granules Quantification

To quantify Htt granules, 3–5 images totaling > 100 cells per sample were acquired using a 20× objective. The granules were analyzed automatically with the Image J plugin “Particle analysis”. A random selection of images was hand-verified. The Htt granule’s average size was analyzed by the Image J software version 1.53C using the ‘analyze particles’ instrument with the threshold “size = 0.00–100.00”.

## 5. Conclusions

This study presents a compelling picture of how coiled-coil motifs drive a cascade of pathological behaviors in Htt exon 1 fragments. These include enhanced aggregation, increased detergent insolubility, interaction with exosomal markers, elevated secretion, and facilitated uptake by other cells. These processes mirror features of prion-like spreading observed in other neurodegenerative diseases and suggest that the coiled-coil domain may be a critical regulator of mHtt propagation. Targeting this structural feature could offer a novel approach to slowing the progression of Huntington’s disease by interfering with the intercellular transfer of toxic Htt species.

A limitation of our study is that our investigations have been performed exclusively in cell lines. Although we confirmed that the aggregation and release of Htt proteins occur in non-neuronal and neuronal cell lines in a similar way, to strengthen our results it will be necessary to perform experiments in more relevant systems, such as animal models of the disease or iPSC-derived neurons obtained from HD patients.

## Figures and Tables

**Table 1:** List of primary antibodies used for biochemical analysis.

Antibody (Clone)	Host Animal	Dilution for Western Blot	Distributor, Catalog Number
PolyQ (3B5H10)	Monoclonal Mouse	1:1000	Sigma, P1874
Huntingtin (EM-48)	Monoclonal Mouse	1:500	Merck Millipore, MAB5374
GAPDH (FL-335)	Polyclonal Rabbit	1:1000	Santacruz, sc-25778

**Table 2:** List of secondary antibodies used for biochemical analysis.

Antibody (Clone)	Host Animal	Dilution for Western Blot	Distributor, Catalog Number
Anti HRP	Mouse	1:10,000	Jackson Immunoresearch, #115-035-174
Anti HRP	Rabbit	1:10,000	GE Healthcare, NA934

## Data Availability

All relevant data are contained within the article. The original contributions presented in the study are included in the article/Supplementary Material; further inquiries can be directed to the corresponding author.

## Supplementary Materials

The following supporting information can be downloaded at https://www.mdpi.com/article/10.3390/ijms26178162/s1.
