# Supplementary figures and images for: Coiled-Coil Structures Mediate the Intercellular Propagation of Huntingtin
Source: Int J Mol Sci. 2025 Aug 22;26(17):8162. doi: 10.3390/ijms26178162 (PMC12427799; doi:10.3390/ijms26178162)

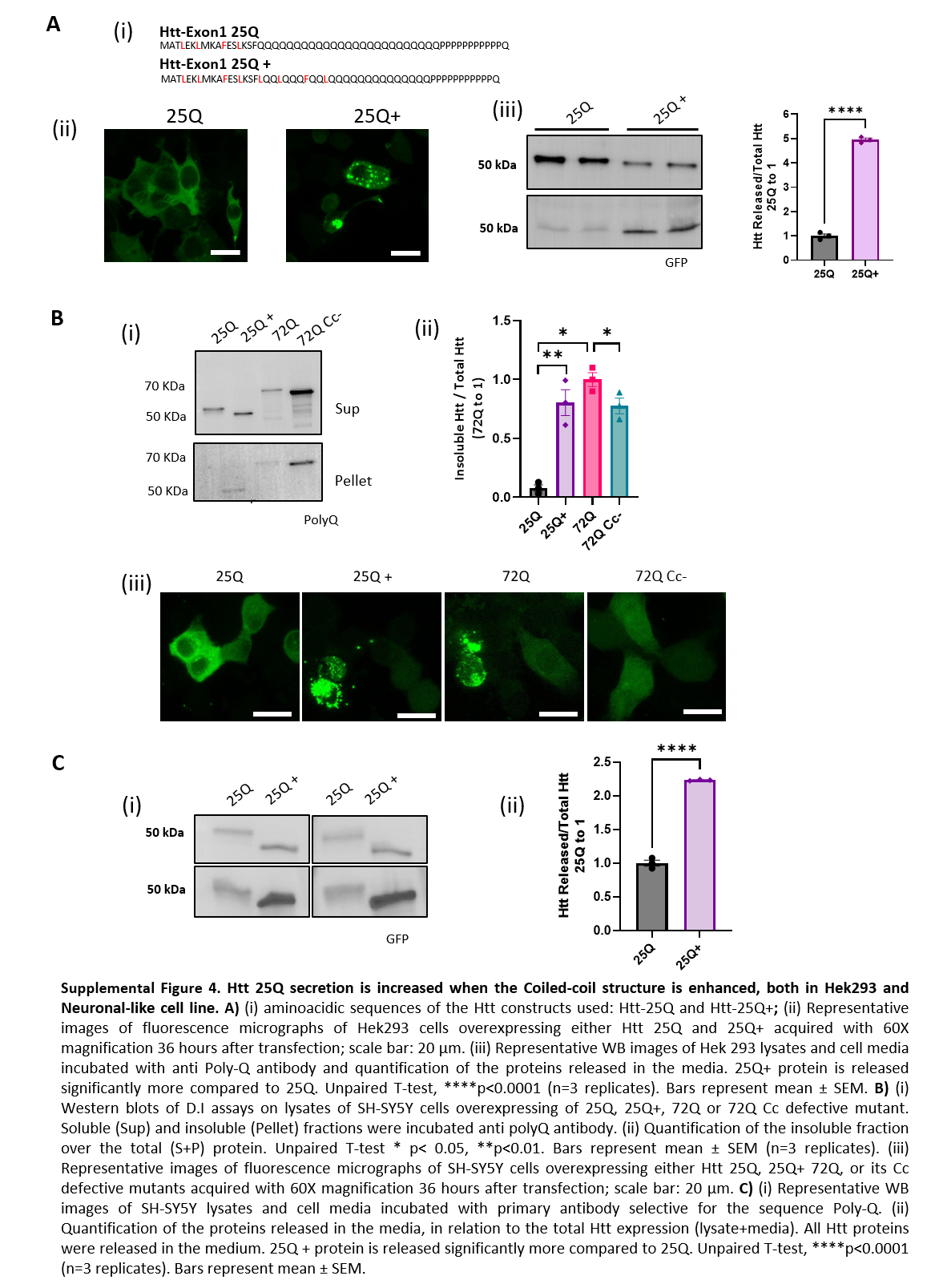

Supplement: Supplementary file 1 [file ijms-26-08162-s001.zip › ijms-3689582_SupplementalFigure_4.png]

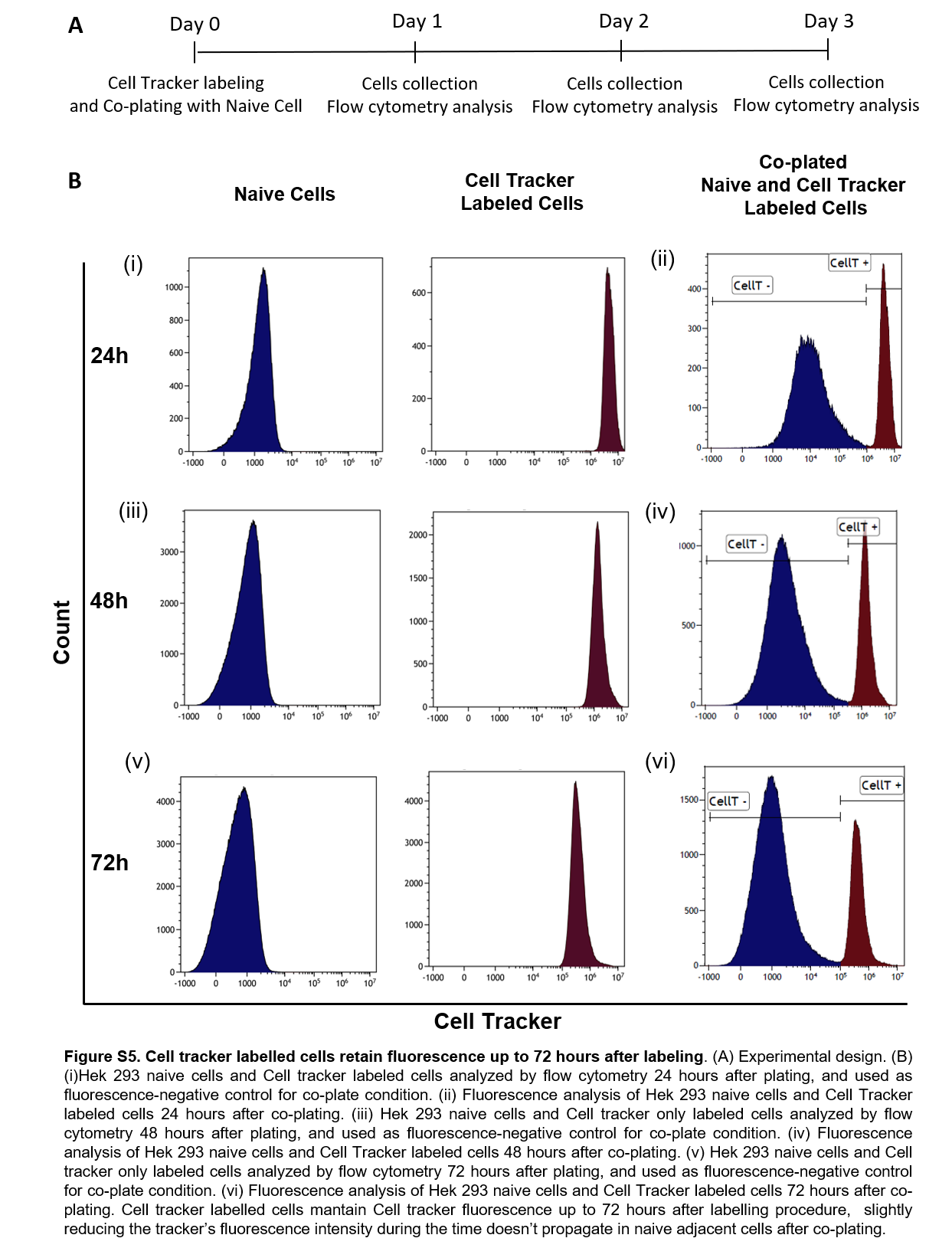

Supplement: Supplementary file 1 [file ijms-26-08162-s001.zip › ijms-3689582_SupplementalFigure_5.png]

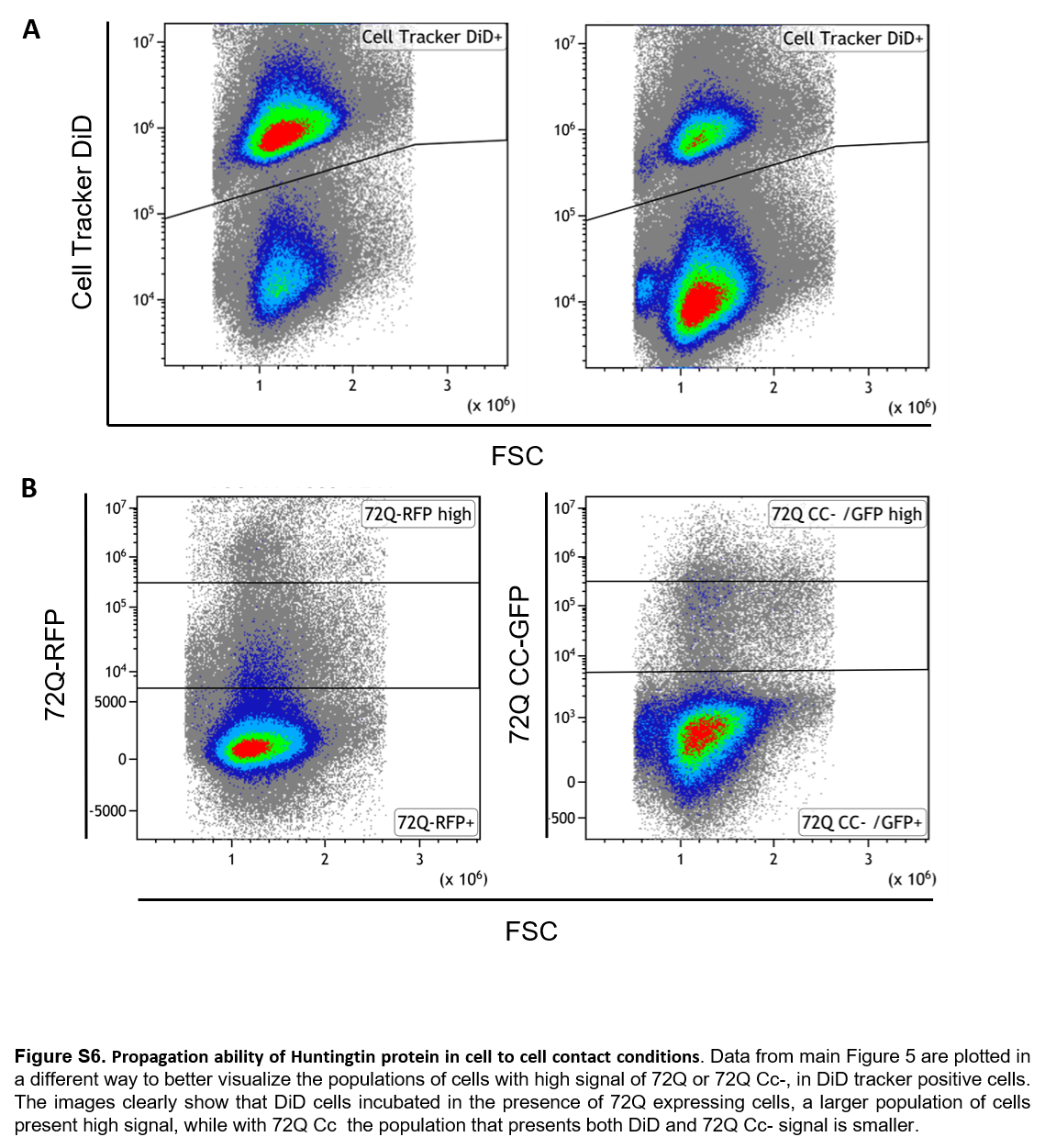

Supplement: Supplementary file 1 [file ijms-26-08162-s001.zip › ijms-3689582_SupplementalFigure_6.png]

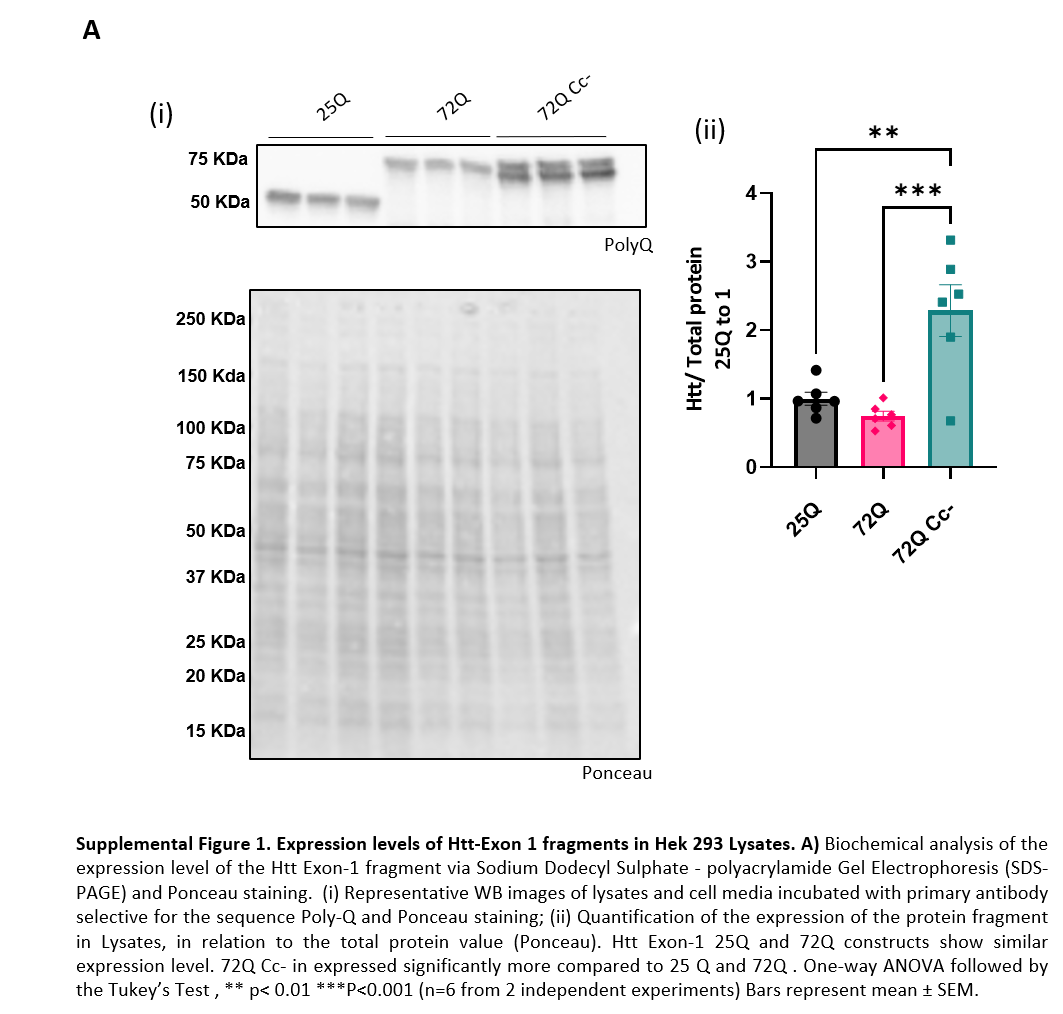

Supplement: Supplementary file 1 [file ijms-26-08162-s001.zip › ijms-3689582_SupplementalFigure_1.png]

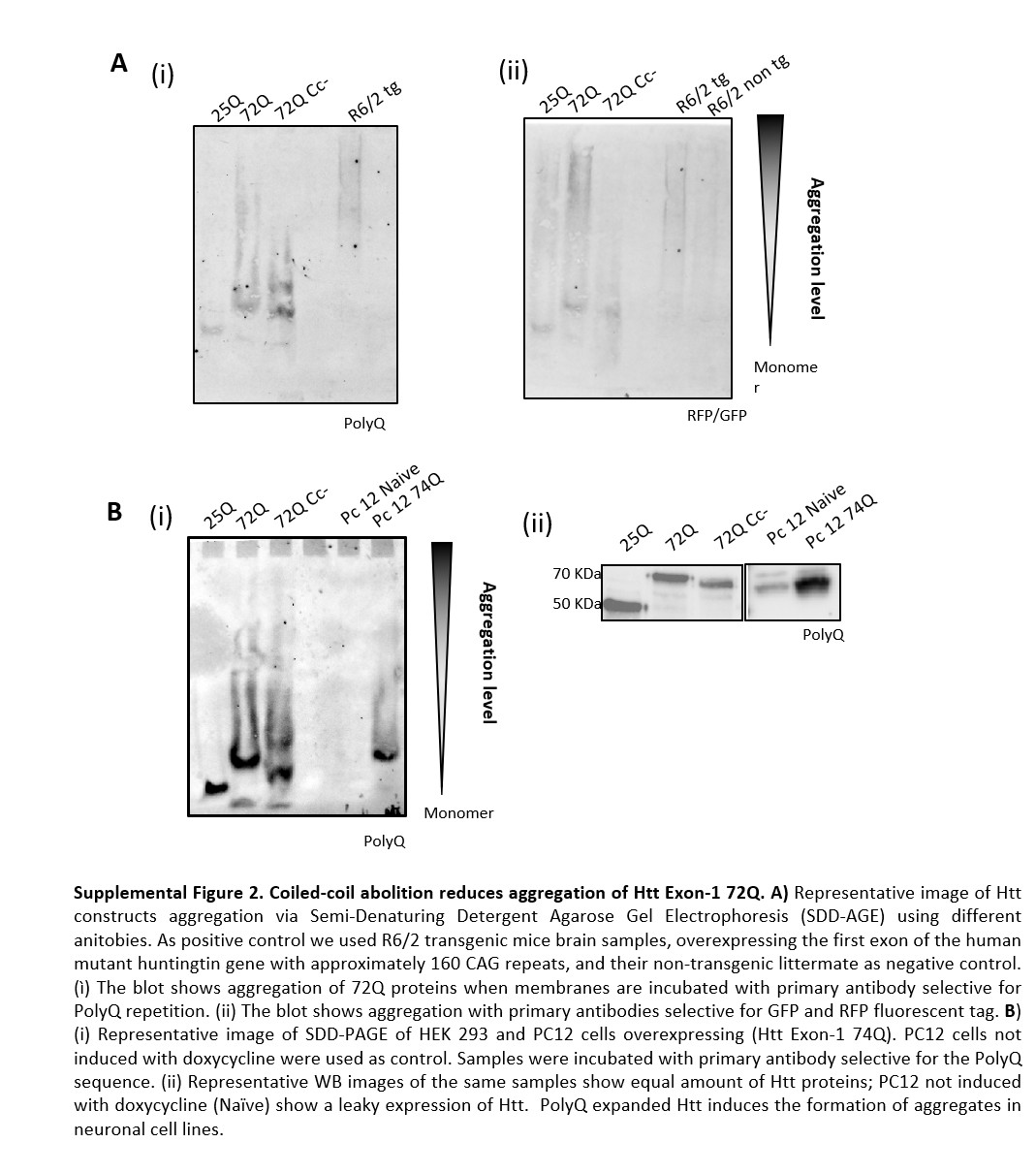

Supplement: Supplementary file 1 [file ijms-26-08162-s001.zip › ijms-3689582_SupplementalFigure_2.png]

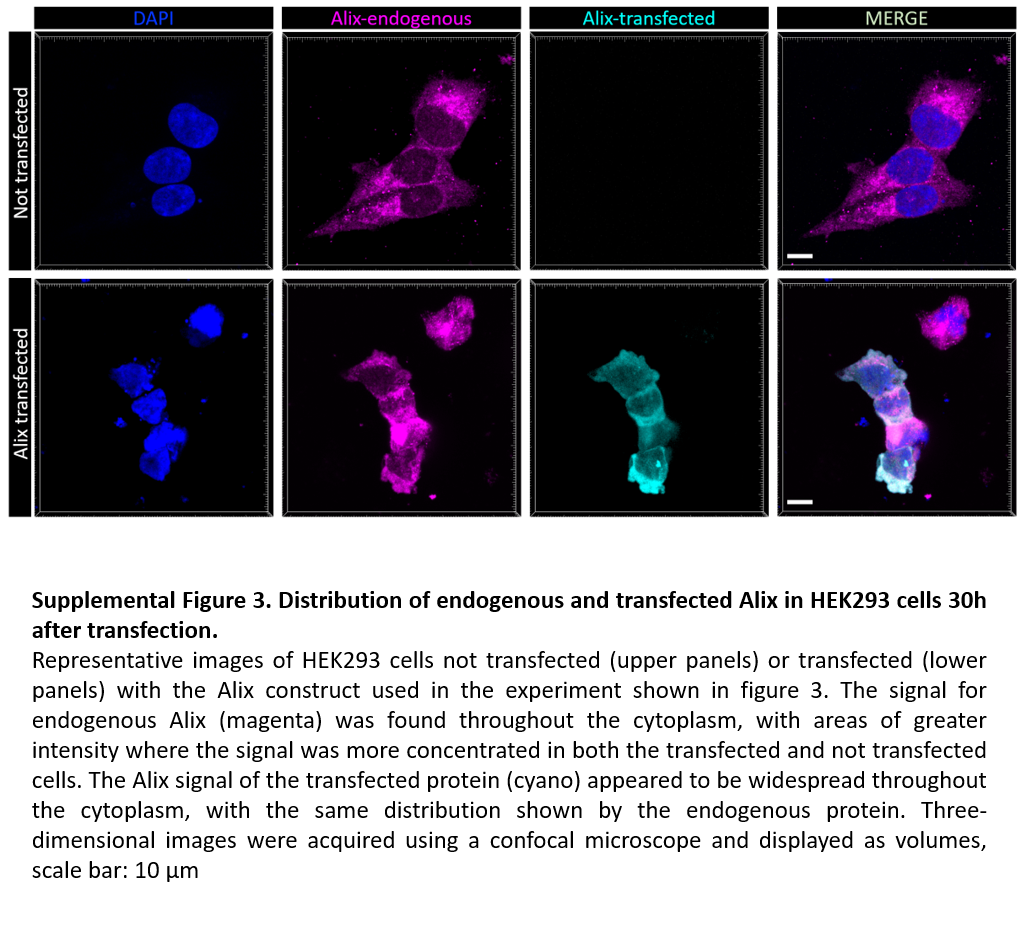

Supplement: Supplementary file 1 [file ijms-26-08162-s001.zip › ijms-3689582_SupplementalFigure_3.png]
